# Supplementary material for: Disentangling the role of NAc D1 and D2 cells in hedonic eating
Source: Mol Psychiatry. 2023 Jul 4;28(8):3531–47. doi: 10.1038/s41380-023-02131-x (PMC10618099; doi:10.1038/s41380-023-02131-x)
Supplement: Supplementary file 1 — Supplementary Figures [file 41380_2023_2131_MOESM1_ESM.docx]

ORIGINAL ARTICLE

**Disentangling the role of NAc D1 and D2 cells in hedonic eating**

Mathilde C. C. Guillaumin^1^, Paulius Viskaitis^1^, Ed Bracey^1^, Denis Burdakov^1^ and Daria Peleg-​Raibstein^1^

^1^Institute for Neuroscience, Department of Health Sciences and Technology, Swiss Federal Institute of Technology, ETH Zurich, 8603 Schwerzenbach, Switzerland

Corresponding author: Daria Peleg-Raibstein, daria-peleg@ethz.ch

**Supplementary figures**

**
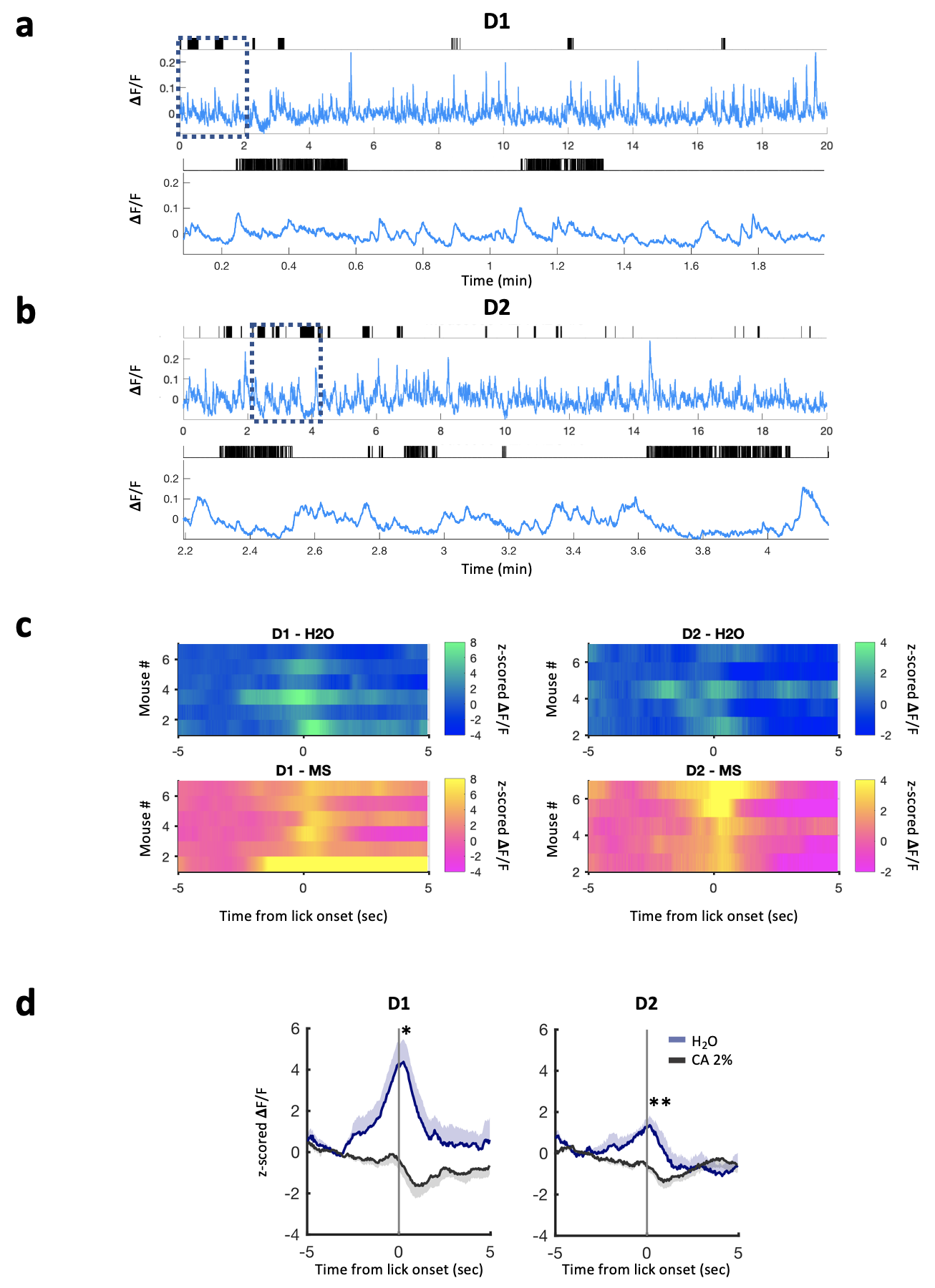
**

**Figure S1. An increase in palatability is reflected in D1 and D2 cell activity.**

(a) Example photometry recording from a 20-min session with access to water from a D1-GCaMP6s mouse. Licks indicated by black raster plot above. Bottom, zoom of photometry signal and licks showing only 2 min indicated by black dotted square above. Photometry signals are (465nm signal – fitted 405 signal)/fitted 405nm signal.

(b) As in (a) for a D2-GCaMP6s mouse.

(c) Heatmaps of the photometry signals of individual mice around water (H2O, blue) or milkshake (MS, pink) lick onset, averaged over all licking bouts for each given mouse from the D1-GCaMP6s (left, n=6) and D2-GCaMP6s (right, n=5) cohorts. For the MS plots, only data from the first milkshake session (MS1) is presented.

(d) Photometry signals around water (H_2_O, blue) or 2% citric acid (CA, black) lick onset, averaged over all licking bouts in D1-GCaMP6s mice (left, n=6) and D2-GCaMP6s mice (right, n=5). Mean across bouts and animals is shown, shaded area represents the SEM. Paired-samples t-test: D1-GCaMP6s mice, delta min-max post lick onset H_2_O vs. CA (t(5)=3.155, p=0.025), max post lick onset H_2_O vs. CA (t(5)=3.866, p=0.012), min post lick onset H_2_O vs. CA (t(5)=2.813, p=0.037); D2-GCaMP6s mice, delta min-max post lick onset H_2_O vs. CA (t(4)=4.364, p=0.012), max post lick onset H_2_O vs. CA (t(4)=6.704, p=0.003), min post lick onset H_2_O vs. CA (t(4)=-0.490, p=0.650).

MS: Milkshake. P-values reported on the figures as follows: *p≤0.05, **p≤0.01, ***p<0.001.


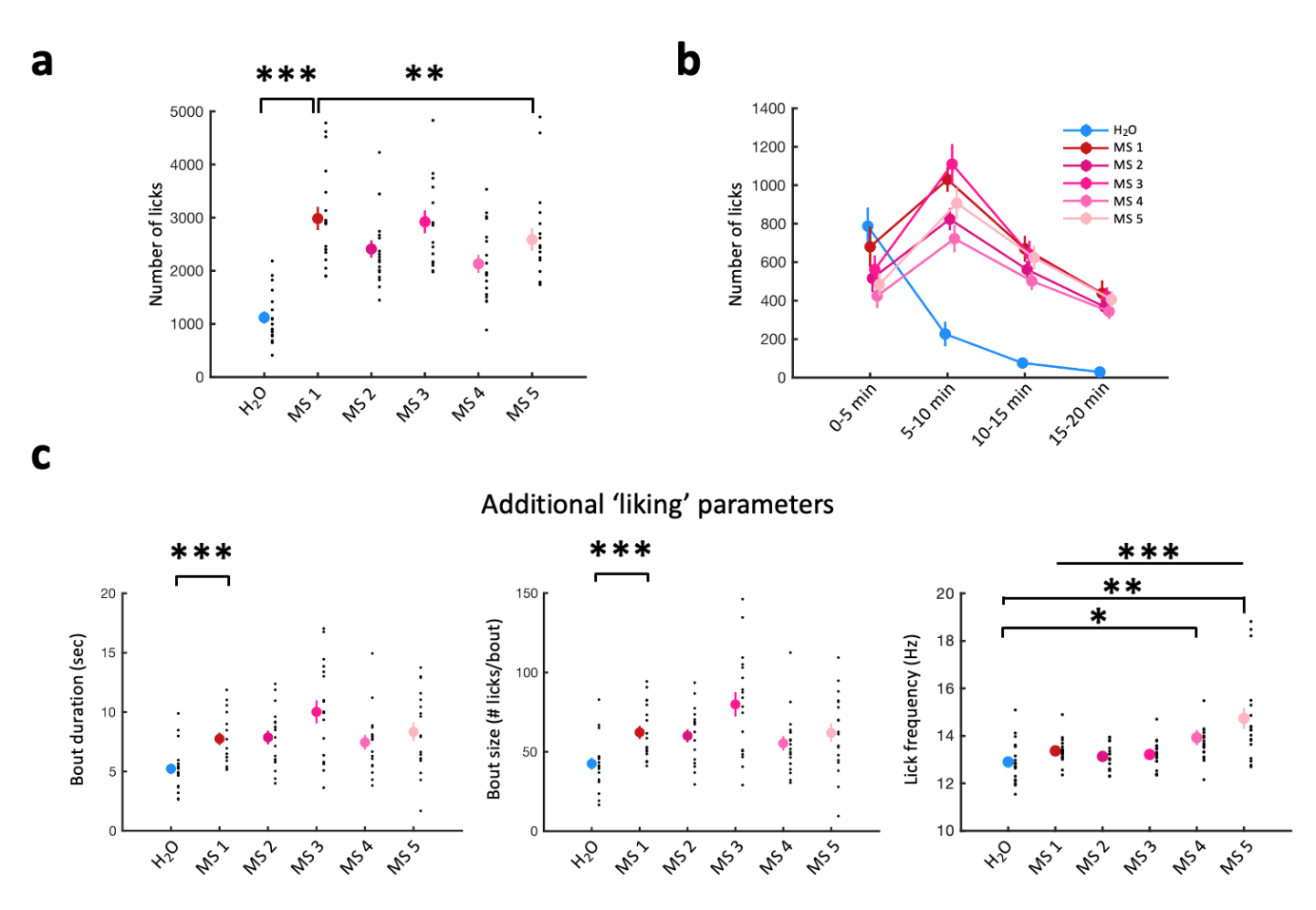


**Figure S2. Additional parameters for the lick macro- and microstructure analysis of the free-consumption task.**

(a) Lick number during daily 20-min sessions with access to water (H_2_O, first session, blue dot) and milkshake (MS - 5 sessions, red and pink dots). Mean ± SEM values are plotted in color, and individual values from each mouse in black. D1-GCaMP6s and D2-GCaMP6s mice are pooled, n=18. One-way repeated measures ANOVA: water vs. MS 1 (F(1,17)=147.327, p<0.001), MS 1 to MS.5 (F(4,68)=19.895, p<0.001), MS 1 vs. MS 5 (F(1,17)=11.006, p=0.004).

(b) Lick number over 5-min blocks across daily 20-min sessions with access to water (first session) and MS (5 sessions). Values are plotted (in color) as mean ± SEM, n=18.

(c) Additional parameters calculated from lick recordings canonically associated with ‘liking’: bout duration (left), bout size (i.e., number of licks per bout, middle) and lick frequency (right) across one session with access to water and 5 sessions with access to MS. D1-GCaMP6s and D2-GCaMP6s mice are pooled. Mean ± SEM values are plotted in color, and individual values from each mouse in black, n=18. One-way repeated measures ANOVA, Bout duration: water vs. MS 1 (F(1,17)=25.529, p<0.001), MS 1 to MS 5 (F(4,68)=4.245, p=0.004), MS 1 vs. MS 5 (F(1,17)=0.675, p=0.423); Bout size: water vs. MS 1 (F(1,17)=26.825, p<0.001), MS 1 to MS 5 (F(4,68)=5.282, p=0.001), MS 1 vs. MS 5 (F(1,17)=0.003, p=0.956); Lick frequency: water vs. MS 4 (F(1,17)=5.651, p=0.029), water vs. MS 5 (F(1,17)=10.813, p=0.004), MS 1 to MS 5 (F(4,68)=6.013, p<0.001).

MS: Milkshake. P-values reported on the figures as follows: *p≤0.05, **p≤0.01, ***p<0.001.


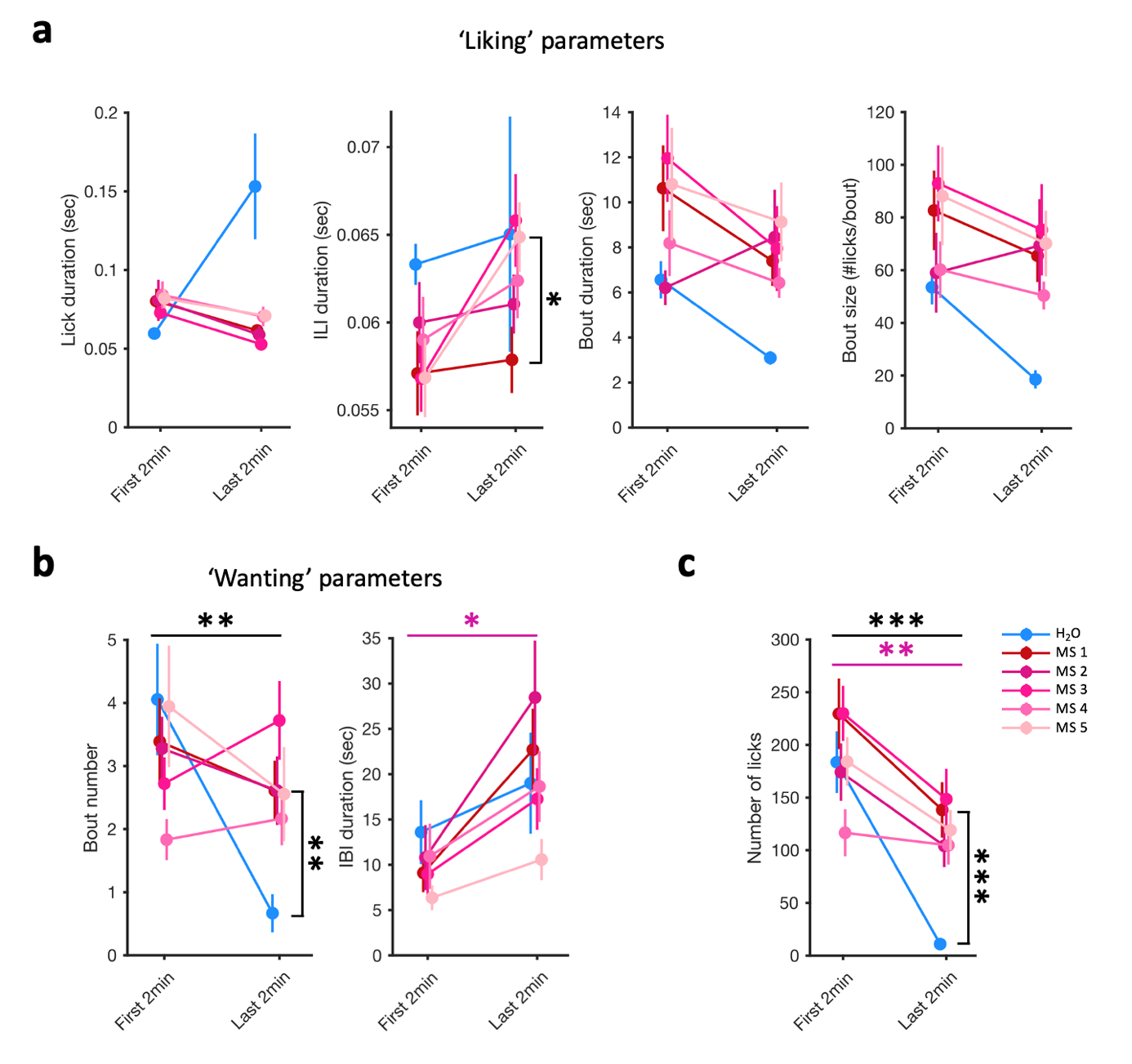


**Figure S3. Changes in lick microstructure parameters within and across sessions with access to water or a palatable food reward.**

(a) ‘Liking’ parameters during the first and last 2min of each daily 20-min session with access to water (H_2_O, first session, blue dot) and milkshake (MS - 5 sessions, red and pink dots). Mean ± SEM values are plotted in color, and individual values from each mouse in black. D1-GCaMP6s and D2-GCaMP6s mice are pooled, n=18. Lick duration: repeated measures ANOVA (time point, drink): N.S. Inter-lick interval (ILI) duration: RM ANOVA (time point, session): MS1 vs. MS5 (session: F(1,10)=7.765, p=0.019; pairwise comparison at Last 2min, MS1 vs. MS5 p=0.045). Bout duration: N.S. Bout size: N.S.

(b) As in (a) but for ‘wanting’ parameters. Bout number: RM ANOVA water vs. MS1 (time point: F(1,17)=9.650, p=0.006, time point*drink: F(1,17)=8.830, p=0.009; for water, First 2min vs. Last 2min: p<0.001; for Last 2min, water vs. MS1: p=0.003). Inter-bout interval (IBI) duration: RM ANOVA MS1 to MS5 (time point: F(1,2)=33.937, p=0.028, pink star).

(c) As in (a) but for the number of licks. RM ANOVA water vs MS1 (time point: F(1,17)=14.978, p=0.001, drink: F(1,17)= 20.624, p<0.001, time point*drink: F(1,17)=4.394, p=0.05; for water, First 2min vs. Last 2min: p<0.001; for Last 2min, water vs. MS1: p<0.001), RM ANOVA MS1 to MS5 (time point: F(1,17)=11.229, p=0.004, pink star, session MS: F(4,68)=5.682, p<0.001).

IBI: inter-bout interval, ILI: inter-lick interval, MS: milkshake. p-values reported on the figures as follows: *p≤0.05, **p<0.01, ***p<0.001.

**
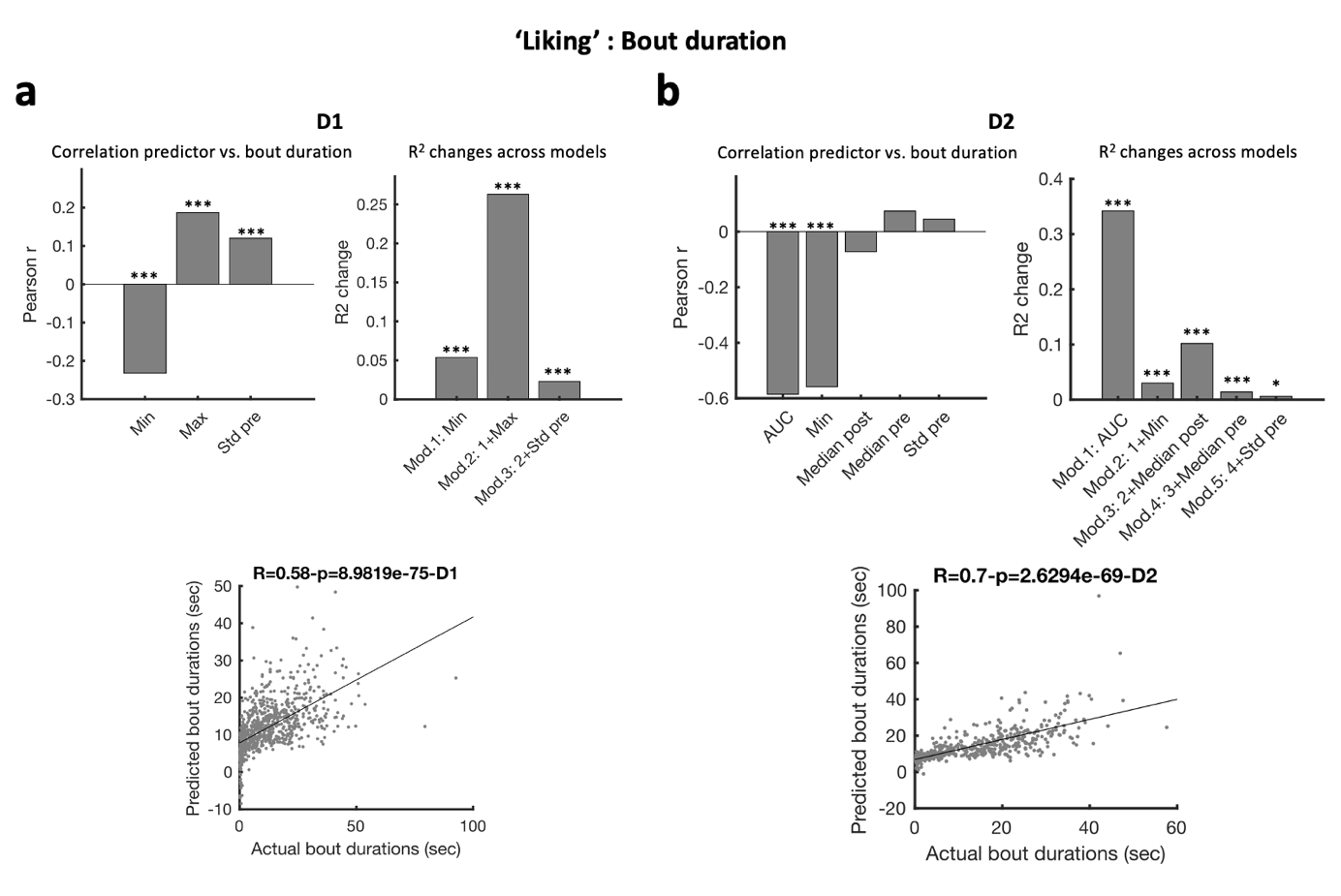
**

**Figure S4. Photometry signal features of NAc D1 and D2 cells around *ad libitum* palatable food consumption are good predictors of ‘liking’ of the food consumed.**

(a) Multiple regression analysis using variables extracted from photometry signals for D1-GCaMP6s mice (n=6) around licking onset to predict licking bout duration. Top left: Pearson’s r values of single correlations between predictors and bout duration (Min: Minimum value in the 5 sec following lick onset, Max: Maximum value in the 5 sec following lick onset, Std: standard deviation of the non-z-scored signal in the 5 sec preceding lick onset – see Methods). Top right: R^2^ change evaluating whether a given regression model (Mod.) significantly improves the prediction of bout duration compared to the previous model; the p-value associated with the R^2^ change are reported above each bar. The R^2^ change indicates whether the amount of variance in the outcome (bout duration) can be explained by the added predictor. For model 1, Min was the only predictor included. The R^2^ change shows that using the Min to predict bout duration significantly improves predictions compared to using the grand mean of bout duration. For model 2, Min and Max were included as predictors. The R^2^ change shows that including the Max of the photometry signal around licking onset in addition to the Min significantly improves the model’s performance in predicting bout duration, with similar reasoning for subsequent models (Model 3). Bottom: predicted vs. actual bout duration, using the final selected regression model (coefficients and equation reported in **Figure 2a**).

(b) As in (a) but for D2-GCaMP6s mice (n=5). AUC: area under the curve over the 5 sec following lick onset, Median pre: median signal over the 3 sec before lick onset, Median post: median signal over the 3 sec following lick onset.

AUC: area under the curve, Max: maximum, Min: minimum, pre: before licking onset, post: following licking onset, Std: standard deviation. p-values reported on the figures as follows: *p≤0.05, ***p<0.001.


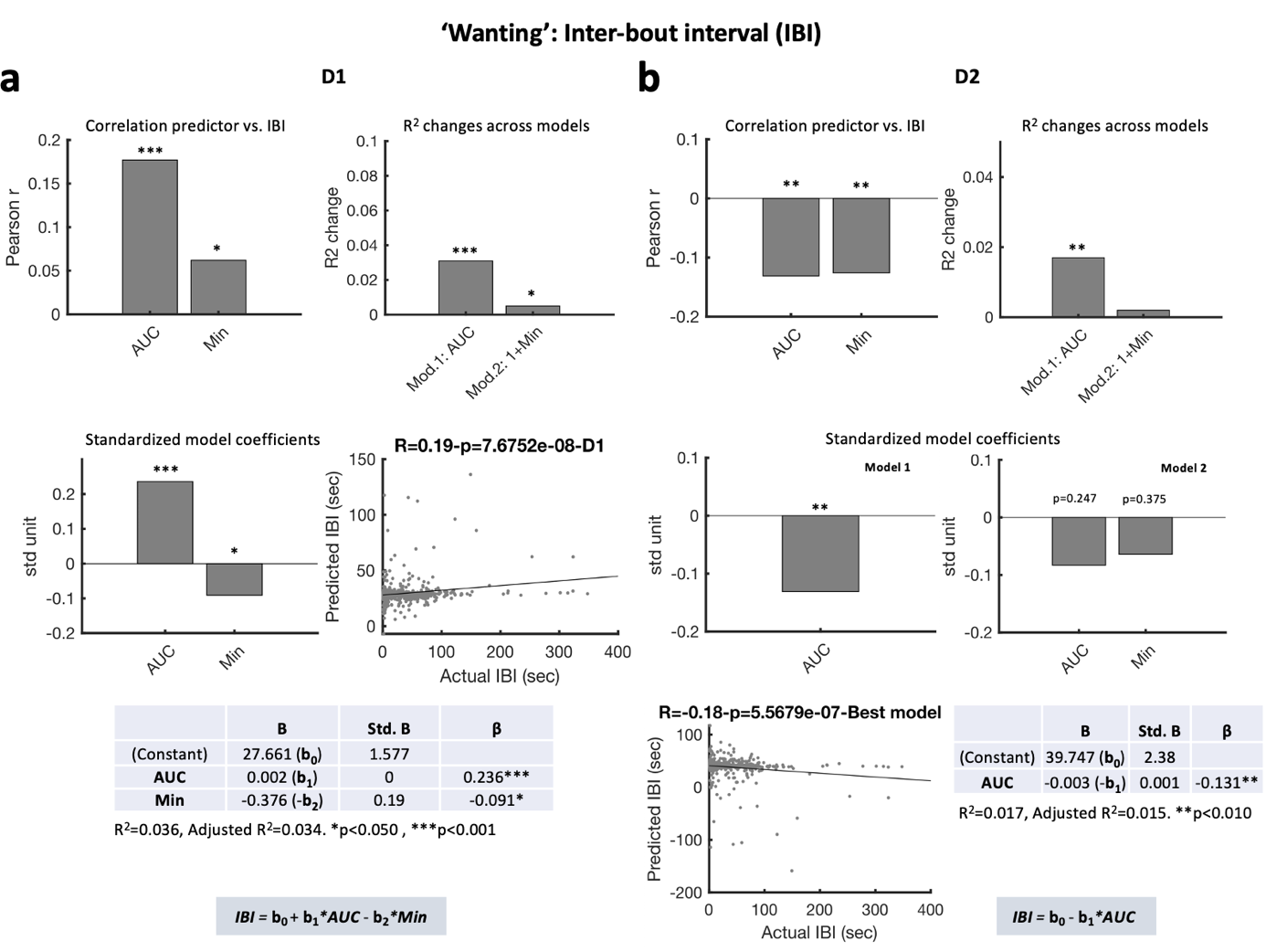


**Figure S5.** **Photometry signal features of NAc D1 and D2 cells around *ad libitum* palatable food consumption are not good predictors of ‘wanting’ of the food consumed.**

(a) As in Figures 2a and S4a but for prediction of inter-bout intervals (IBI) in D1-GCaMP6s mice (n=6).

(b) As in (a) but for D2-GCaMP6s mice (n=5). Note that here although both the AUC and Min predictors were individually significantly correlated with IBI (top left), only the model considering the AUC yielded a significant improvement in predicting IBI duration compared to using the grand mean of IBI duration (i.e., no better predicting power of model 2 compared to model 1) (top right and middle right).

AUC: area under the curve, IBI: inter-bout interval, Min: minimum, Std: standard deviation. p-values reported on the figures as follows: *p≤0.05, **p≤0.01, ***p<0.001.

**
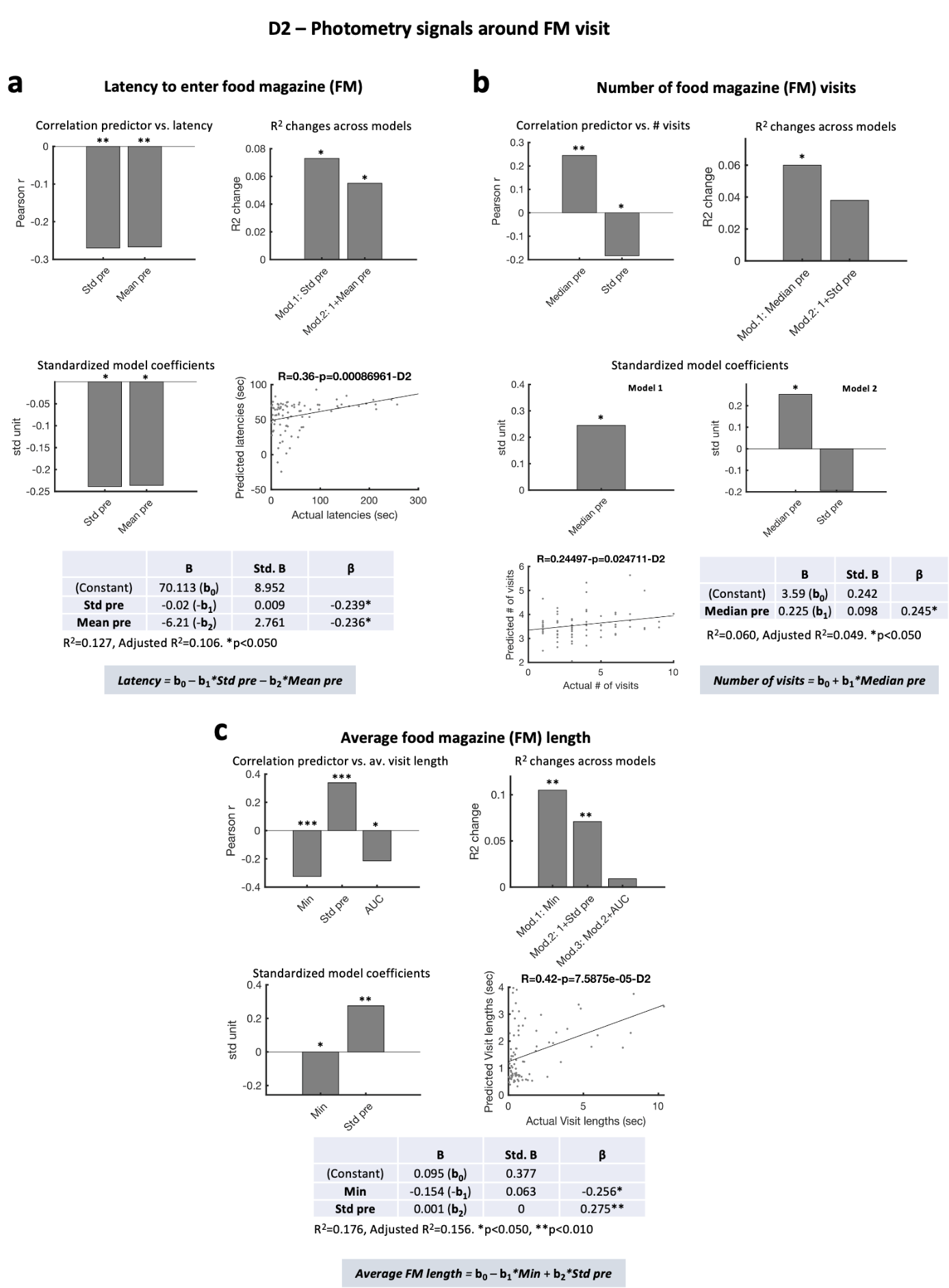
**

**Figure S6. The decrease in D2 cell activity during consumption favors longer licking bouts.**

(a) Multiple regression analysis using variables extracted from the photometry signals for D2-GCaMP6s mice (n=5) around food magazine (FM) visit onsets to predict the latency to enter the FM following a food delivery. Top left: Pearson’s r values of single correlations between predictors and FM visit latency (Mean pre: mean signal over the 3 sec before FM visit onset, Std pre: standard deviation of the non-z-scored signal over the 5 sec preceding FM visit onset – see Methods). Top right: R^2^ change evaluating whether a given regression model (Mod.) significantly improves the prediction of FM visit latency compared to the previous model; the p-value associated with the R^2^ change is reported above each bar. The R^2^ change indicates whether the amount of variance in the outcome (latency to enter the FM) can be explained by the added predictor. For model 1, Std pre was the only predictor included. The R^2^ change shows that using the Std pre to predict latency significantly improves predictions compared to using the grand mean of latencies. For model 2, Std pre and Mean pre were included as predictors. The R^2^ change shows that including the mean photometry signal over the 3 sec before FM visit in addition to the Std pre significantly improves the model’s performance in predicting latency to enter the FM. Middle left: Standardized coefficients of the final model; standardized coefficients allow to compare the relative contribution of each predictor, regardless of the units used to measure them. The higher the absolute value of a standardized coefficient, the higher the contribution to the model. The p-values associated with these contributions (i.e., whether they are significant, regardless of their magnitude) is reported above each bar. Middle right: Plot of the actual vs. predicted FM visit latency with the R and p values reported on top. Bottom: model coefficients (B), standard deviation of the coefficients (Std. B) and standardized model coefficient (β) of each predictor in the final model, of which the equation is reported below the table.

(b) As in (a) but for prediction of number of food magazine visits. Median pre: median signal over the 3 sec before FM visit onset. Note that here although both the Median pre and Std pre predictors were individually significantly correlated with the number of FM visits (top left), only the model considering the Median pre yielded a significant improvement in predicting number of FM visits compared to using the grand mean of the number of visits (i.e., no better predicting power of model 2 compared to model 1) (top right and middle right).

(c) As in (a) but for the average food magazine visit length. Min and AUC: Minimum value and area under the curve in the 5 sec following FM visit onset.

AUC: area under the curve. FM: food magazine, Min: minimum, pre: before FM visit onset, Std: standard deviation. p-values reported on the figures as follows: *p≤0.05, **p≤0.01, ***p<0.001.

**
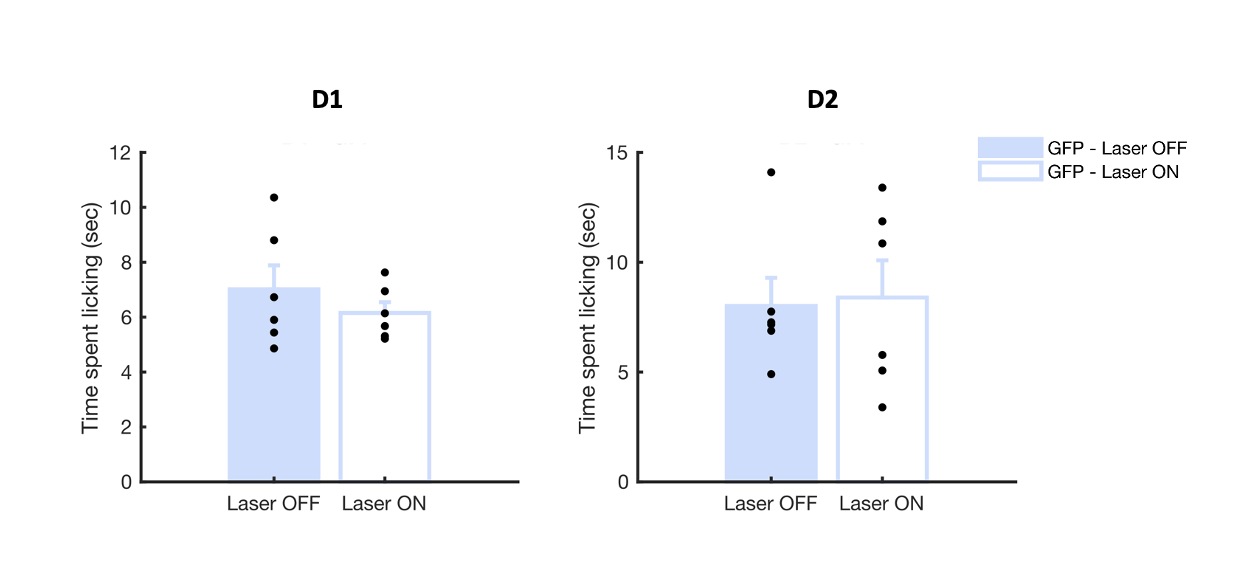
**

**Figure S7. Laser effect on free-licking in GFP mice.**

Difference in the time spent licking during a free-licking paradigm where Laser was OFF for 3 min and ON for 3 min (pattern of activation as described in Methods) in D1-cre (left, n=6) and D2(A2a)-cre mice (right, n=6) expressing GFP. One-way ANOVA (laser condition), D1: F(1,5)=2.181, p=0.200, D2: F(1,5)=0.090, p=0.777.

**
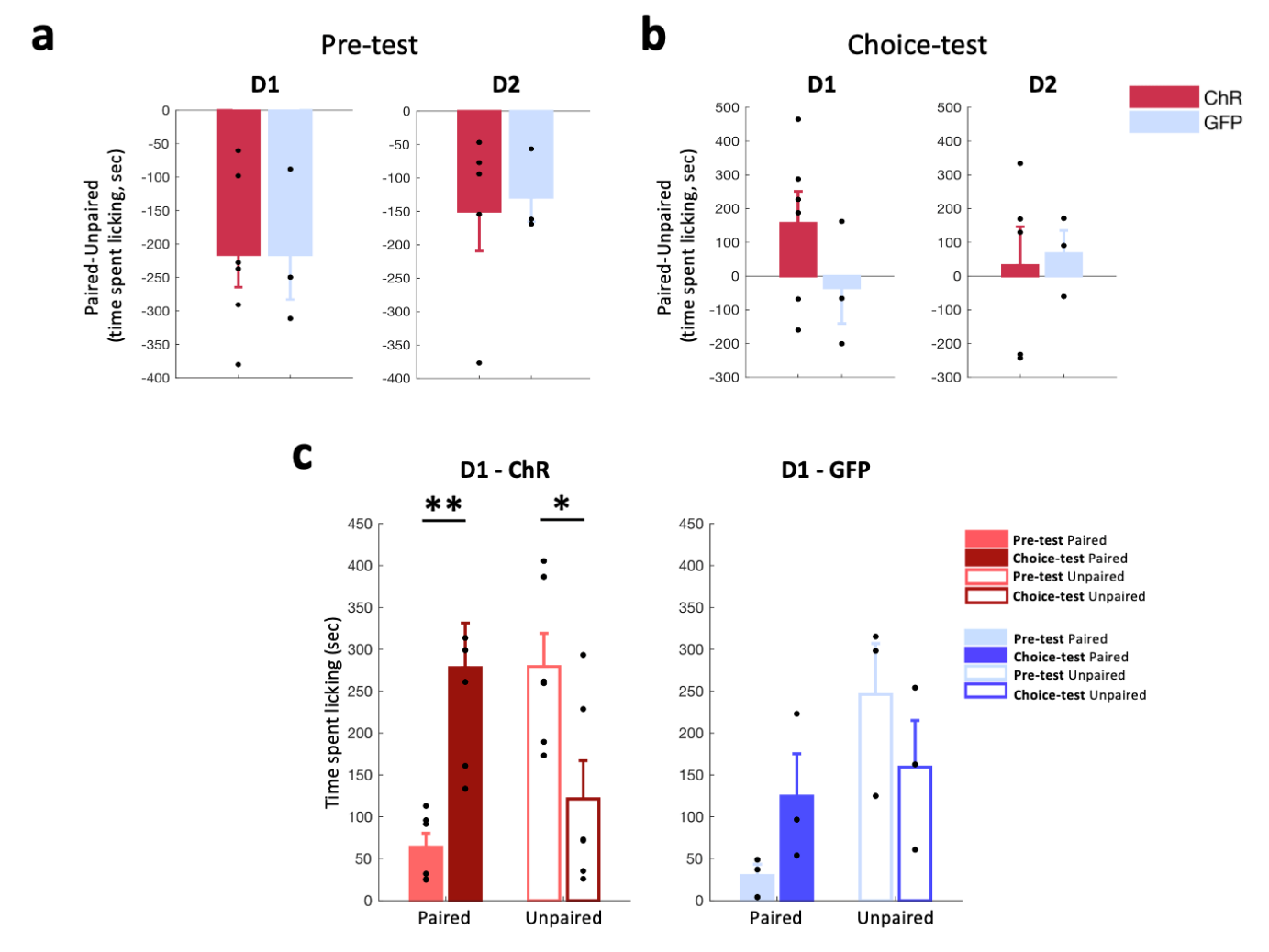
**

**Figure S8. Changes in time spent licking the paired and unpaired flavours in the pre-test and choice-test for D1-cre and D2(A2a)-cre mice.**

(a) Difference in the time spent licking the paired and unpaired flavors during the pre-test session of the hedonic shifting experiment in D1-cre (left) and D2(A2a)-cre mice (right) expressing ChrimsonR (ChR, red, D1-ChR: n=6, D2-ChR: n=5) or GFP (blue, D1-GFP: n=3, D2-GFP: n=3). One-way ANOVA (opsin), D1: F(1,7)<0.001, p=0.994, D2: F(1,6)=0.062, p=0.812.

(b) As in (a) but for the time spent licking each flavor on the choice-test session. One-way ANOVA (opsin), D1: F(1,7)=1.526, p=0.257, D2: F(1,6)=0.048, p=0.833.

(c) Difference across the pre-test and choice-test sessions in the time spent licking the paired and unpaired flavours in D1-ChR (left) and D1-GFP (right) mice. D1-ChR: two-way RM ANOVA (test day, flavor): test day*flavor, F(1,5)=22.604, p=0.005; Paired flavor, pre-test vs. choice-test, F(1,5)=19.069, p=0.007; Unpaired flavor, pre-test vs. choice-test, F(1,5)=12.919, p=0.016. D1-GFP: two-way RM ANOVA (test day, flavor): test day*flavor, F(1,2)=1.153, p=0.395; Paired flavor, pre-test vs. choice-test, F(1,2)=2.269, p=0.271; Unpaired flavor, pre-test vs. choice-test, F(1,12)=0.588, p=0.523.

p-values reported on the figures as follows: *p≤0.05, **p<0.01.

**
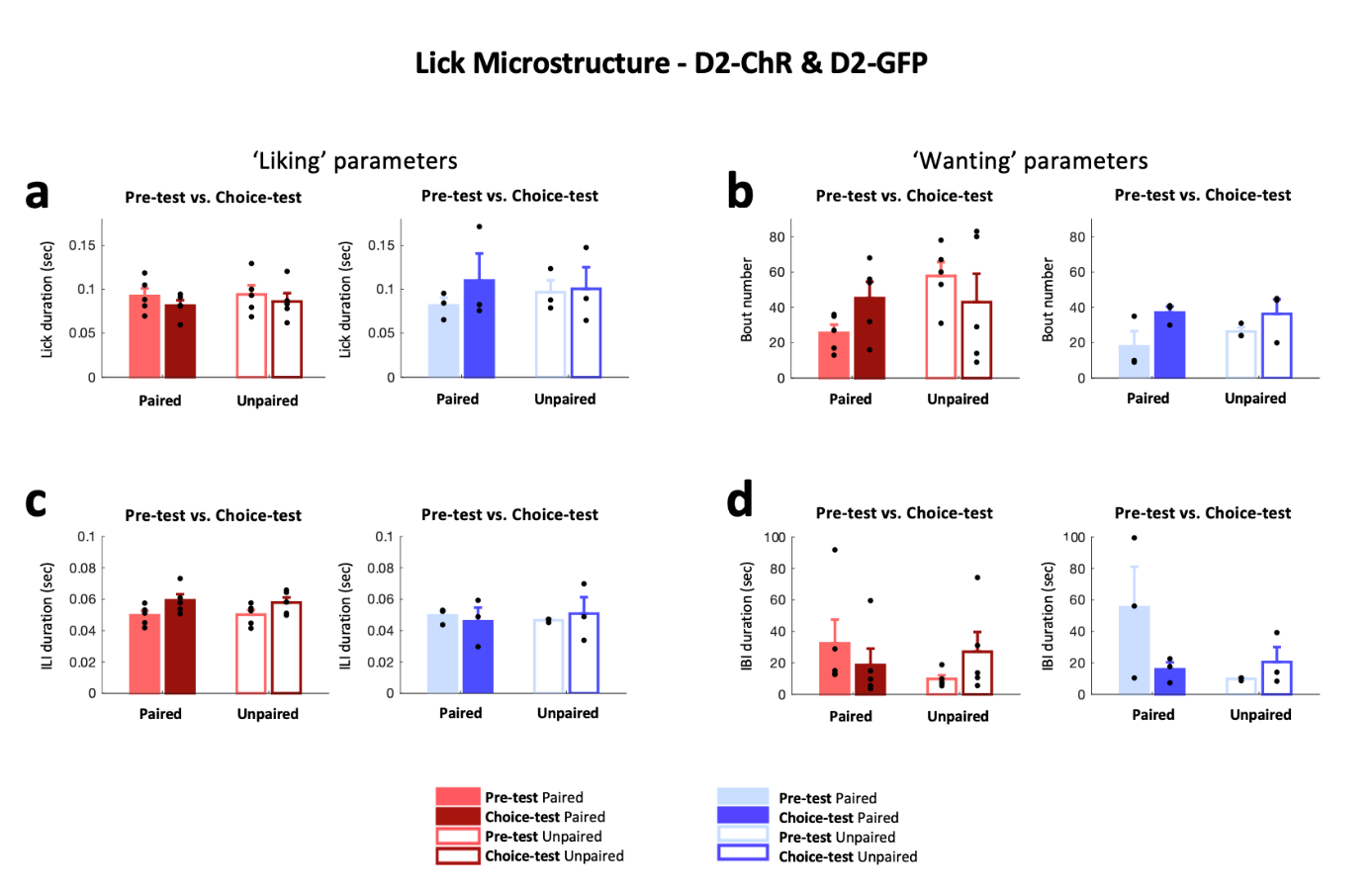
**

**Figure S9. Activation of D2 cells does not lead to a shift in flavor preference for the optoactivation-paired flavor.**

(a) Single-lick duration during the pre-test & choice-test sessions in the paired (full bars) and unpaired (empty bars) flavors in D2-ChR (left, red, n=5) and D2-GFP (right, blue, n=3) mice. Mean ± SEM values are plotted in color, and individual values from each mouse in black. RM-ANOVA: Choice-test vs. pre-test, D2-ChR: paired flavor (test day: F(1,4)=1.307, N.S.), unpaired flavor (test day: F(1,4)=0.599, N.S.); D2-GFP: paired flavor (test day: F(1,2)=0.517, N.S.), unpaired flavor (test day: F(1,2)=0.011, N.S.).

(b) As in (a) but for the number of licking bouts. RM-ANOVA: Choice-test vs. pre-test, D2-ChR: paired flavor (test day: F(1,4)=2.538, N.S.), unpaired flavor (test day: F(1,4)=0.423, N.S.); D2-GFP: paired flavor (test day: F(1,2)=7.367, N.S.), unpaired flavor (test day: F(1,2)=0.906, N.S.).

(c) As in (a) but for inter-lick interval (ILI) durations. RM-ANOVA: Choice-test vs. pre-test, D2-ChR: paired flavor (test day: F(1,4)=6.971, N.S.), unpaired flavor (test day: F(1,4)=1.887, N.S.); D2-GFP: paired flavor (test day: F(1,2)=0.141, N.S.), unpaired flavor (test day: F(1,2)=0.153, N.S.).

(d) As in (a) but for the inter-bout interval (IBI) durations. RM-ANOVA: Choice-test vs. pre-test, D2-ChR: paired flavor (test day: F(1,4)=0.394, N.S.), unpaired flavor (test day: F(1,4)=2.593, N.S.); D2-GFP: paired flavor (test day: F(1,2)=2.962, N.S.), unpaired flavor (test day: F(1,2)=1.342, N.S.).

ChR: ChrimsonR, IBI: inter-bout interval, ILI: inter-lick interval.


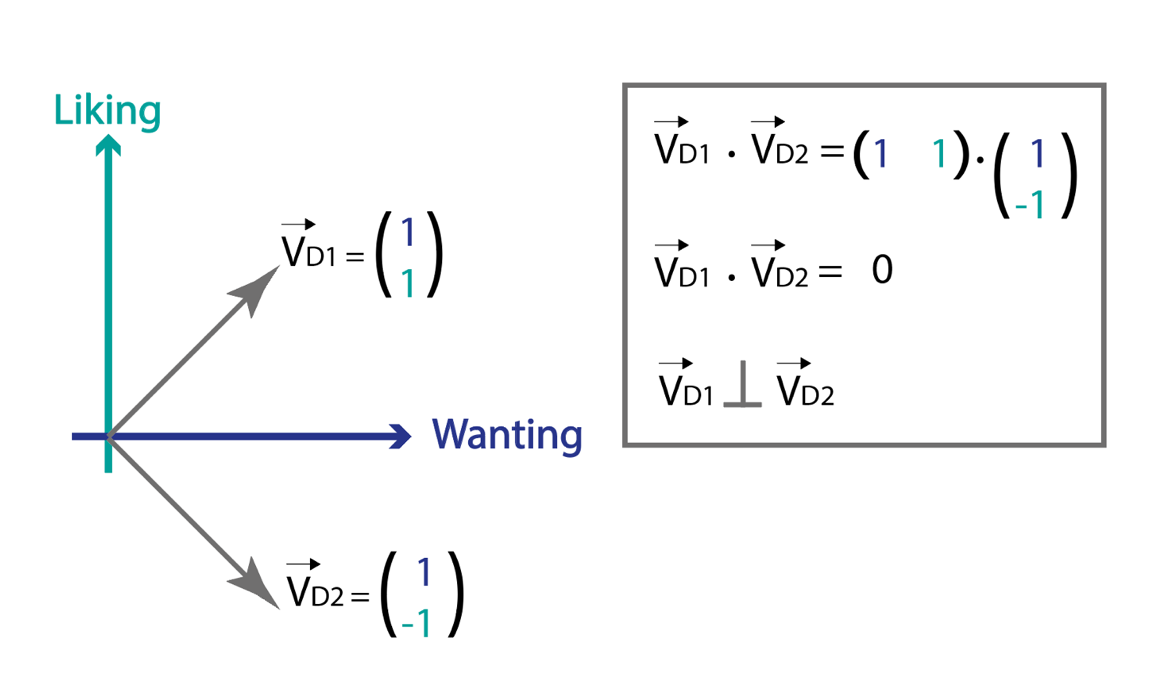


**Figure S10. 2D representation of the effects of D1 and D2 cell activity on ‘liking’ and ‘wanting’.**

Representing ‘liking’ and ‘wanting’ as a 2D space underlying hedonic eating, our data show that D1 and D2 cells, if expressed in a binary manner (0 or 1), are orthogonal in that space and can themselves form a base of a 2D space that can be mapped onto the ‘liking’ and ‘wanting’ behavioral space: D1 cell activity increases ‘wanting’ (x_D1_=1) and ‘liking’ (y_D1_=1), thus $\vec{D1}$ =(1, 1), while D2 cell activity increases ‘wanting’ (x_D2_=1) and decreases ‘liking’ (y_D2_=-1), thus $\vec{D2}$=(1, -1). This would support the idea that evolutionary processes led to these two cell populations that can independently and mutually influence hedonic eating. D1 cells are prone to lead to a maladaptive cycle of increased ‘liking’ and ‘wanting’, thus driving overeating, while D2 cells cannot reinforce both processes and are faced with a tradeoff between increasing ‘wanting’ by being more active or allowing ‘liking’ by remaining silent.
